# Antimicrobial Effect of Chitosan Nanoparticles and *Allium* Species on *Mycobacterium tuberculosis* and Several Other Microorganisms

**DOI:** 10.3390/microorganisms12081605

**Published:** 2024-08-06

**Authors:** Jocelyn Olivas-Flores, José Román Chávez-Méndez, Nydia Alejandra Castillo-Martínez, Héctor Javier Sánchez-Pérez, Aracely Serrano-Medina, José Manuel Cornejo-Bravo

**Affiliations:** 1 Faculty of Chemical Sciences and Engineering, Autonomous University of Baja California, Calzada Universidad 14418, Parque Industrial Internacional, Tijuana 22424, Mexico; olivasj@uabc.edu.mx; 2 Faculty of Health Sciences, Autonomous University of Baja California, Blvd Universitario No. 1000, Valle San Pedro, Tijuana 21500, Mexico; roman.chavez@uabc.edu.mx (J.R.C.-M.); nydia.castillo@uabc.edu.mx (N.A.C.-M.); 3 Department of Health, El Colegio de la Frontera Sur (ECOSUR), Mexican Network for Research in Tuberculosis and Other Mycobacterioses, San Cristóbal de Las Casas 29290, Mexico; hsanchez@ecosur.mx; 4 Faculty of Medicine and Psychology, Autonomous University of Baja California, Calzada Universidad 14418, Parque Industrial Internacional, Tijuana 22424, Mexico

**Keywords:** nanomedicine, chitosan, tuberculosis, antimicrobial activity, garlic extract, antimicrobial resistance

## Abstract

This study evaluates the antimicrobial efficacy of chitosan nanoparticles (CNPs), varying in size, against clinical isolates of *Mycobacterium tuberculosis* (MTB), *E. coli*, *S. aureus*, *E. faecalis*, and *C. albicans*, as well as the antimicrobial effects of aqueous extracts and lyophilized powders of *Allium* (garlic) species. CNPs were synthesized through ionotropic gelation and characterized by Z potential, hydrodynamic diameter (dynamic light scattering, DLS), and SEM. Aqueous garlic extracts were prepared via decoction. We assessed antimicrobial activity using disk diffusion and broth microdilution methods; in addition, a modified agar proportion method in blood agar was used for antimicrobial activity against MTB. CNPs inhibited MTB growth at 300 μg for 116.6 nm particles and 400 μg for 364.4 nm particles. The highest antimicrobial activity was observed against *E. faecalis* with nanoparticles between 200 and 280 nm. *Allium sativum* extract produced inhibition for *C. albicans* at 100 μg. The results indicate that CNPs possess significant antimicrobial properties against a range of pathogens, including MTB, at high concentrations. On the other hand, aqueous *Allium sativum* extracts exhibited antimicrobial activity. Nonetheless, due to their instability in solution, the use of lyophilized *Allium sativum* powder is preferable.

## 1. Introduction

Infections caused by microorganisms represent a significant threat to public health, accounting for more than 400 million life years lost annually worldwide [1]. The emergence of antimicrobial resistance (AMR) by bacteria and fungi is a major global concern, with significant repercussions on both health systems and the countries’ economies. Tuberculosis (TB) presents significant antimicrobial resistance due to its prolonged treatment regimen, ranging from 6 (susceptible cases) to 9 or more months (multidrug-resistant (MDR) cases), as well as associated side effects. These factors have led to problems with adherence and treatment follow-up, increasing the number of MDR-TB and extremely resistant TB (XDR-TB) strains. Although MDR and XDR-TB can be treated with second-line drugs, these are expensive and can lead to adverse effects [2].

Conventional antimicrobials are facing increasing challenges due to the emergence of resistant strains, driving the need to search for therapeutic alternatives. This effort to find new treatment options has sparked substantial interest in the use of nanotechnology and the incorporation of natural products given their promising antimicrobial capacity.

One of the most abundant and studied biopolymers is chitosan (poly-(1,4-β-D-glucopyranosamine). Chitosan is derived from chitin, which is the second most abundant natural polymer after cellulose. Chitin is primarily found in the exoskeletons of crustaceans such as shrimp, crabs, and lobsters, as well as in the cell walls of fungi and some insects. The conversion of chitin to chitosan involves a deacetylation process, where the acetyl groups in chitin are removed, typically using alkaline treatments [3,4]. Chitosan has been widely used in the pharmaceutical field due to its biocompatibility and biodegradability, in addition to showing antimicrobial activity against various microorganisms.

Chitosan has been prepared as nanoparticles by various methods [5]. Chitosan nanoparticles (CNPs) offer significant advantages over regular chitosan, making them a powerful tool in various fields such as medicine, agriculture, and environmental science. Due to their larger surface-area-to-volume ratio, they exhibited enhanced reactivity and interaction with target cells or molecules, improving the effectiveness of applications such as drug delivery and antimicrobial treatments. In addition, they exhibit better solubility and stability in aqueous solutions than bulk chitosan, which is crucial for biomedical applications where consistent and predictable behavior in the body is required (e.g., avoiding the risk of thrombosis). The nanometric size also improves penetration through physiological membranes, enhancing the bioavailability of loaded drugs. CNPs can be easily incorporated into various formulations, including hydrogels, creams, and films, providing greater flexibility for different applications. Due to their biocompatibility and biodegradability, they generally exhibit lower toxicity compared to other synthetic nanoparticles, making them suitable for long-term use within the body [3,4].

It was shown that CNPs form complexes with Mn^2+^, Zn^2+^, Cu^2+^, and Ag^+^, a process that significantly enhanced its antibacterial activity against *Staphylococcus aureus*, *Salmonella enterica* serotype Choleraesuis, and *Escherichia coli* [6,7,8,9]. The antimicrobial activity of low-molecular-weight CNPs at neutral pH against *Streptoccocus mutans* and fungicidal effects are mainly related to the interaction of positively charged chitosan with the negatively charged cell walls or membranes [10,11,12,13].

Similarly, the antibacterial capacity of chitosan nanoformulations against *Mycobacterium tuberculosis* (MTB) strains functioning as a drug delivery system has been reported to improve both their efficacy and administration [14,15]. Furthermore, CNPs have been shown to possess intrinsic antimycobacterial properties, suggesting the potential of chitosan as a source compound for future antimycobacterial drug development [16].

Natural extracts derived from plants, herbs, and other biological sources have long been recognized for their diverse therapeutic properties, including antimicrobial activity. In the last decade, there has been a remarkable increase in the acceptance of and interest in therapies that are based on natural products, both in developing and developed countries, because the use of such products is often more economical and has a lower incidence of side effects compared to synthetic drugs [17,18].

*Allium sativum*, popularly known as garlic, has been reported to exhibit various pharmacological properties. One of the earliest references to medical treatments using garlic is found in the ancient Egyptian Codex Ebers from 1550 B.C. This text includes recommendations for using garlic to treat abnormal growths such as abscesses, circulatory ailments, general malaise, and infestations by insects and parasites. This historical document underscores garlic’s longstanding role in traditional medicine, particularly for addressing various health issues and infections [19]. Garlic was also believed to enhance strength and endurance. In traditional Chinese medicine, garlic is used to improve digestion, treat respiratory issues, and serve as a general health tonic. It is believed to promote cardiovascular health and possesses antimicrobial properties. In European folk medicine, garlic was utilized to treat colds, flu, and infections, as well as to address cardiovascular problems and to lower cholesterol levels [20,21]. According to traditional medical practices of Ayurvedic and Greek medicine, garlic has been recognized as one of the established remedies for tuberculosis [22]. Furthermore, recent reports indicate that the whole extract of *Allium sativum* exhibits superior antimicrobial activity against the Mtb H37Rv strain compared to standard drugs (isoniazid, rifampicin, and ethambutol), which makes it a formidable contender for its further development as an antituberculosis agent [23].

The active search for therapeutic alternatives involves not only the development of new drugs but also the optimization of clinical practices, the promotion of infection prevention, and awareness of the correct use of antibiotics. In terms of public health, early detection of susceptibilities of clinical isolates to antituberculosis drugs is crucial to ensure timely and effective treatment. Therefore, agile, and effective methods are needed to accurately diagnose and rapidly follow up. The most prevalent conventional methods for assessing MTB susceptibility encompass the ratio method, carried out on media such as Lowenstein–Jensen (LJ) medium and Middlebrook 7H10-11 agar, which are characterized as complex and lengthy, and the BACTEC 460 TB system, which, although faster, requires specialized equipment, resulting in increased costs [24]. As an alternative option, it has been reported that blood agar is suitable for the growth and incubation of mycobacteria, including those responsible for TB. In addition, its usefulness for culture and susceptibility testing of first-line drugs has been evidenced [25,26,27,28].

In this study, the antimicrobial potential of CNPs with size variations and the aqueous extract and lyophilized powder of three *Allium* species against various microorganisms, including Gram-positive bacteria, Gram-negative bacteria, a fungus, and clinical isolates of *Mycobacterium tuberculosis*, were investigated to determine whether these materials could be effective in combating infectious diseases. In addition, an innovation to growth media was implemented to improve the efficiency of susceptibility testing for MBT.

## 2. Materials and Methods

Low-molecular-weight chitosan (91.7% deacetylated), sodium chloride (NaCl), sodium pentabasic tripolyphosphate (TPP), sodium hydroxide (NaOH), and glacial acetic acid were purchased from Sigma-Aldrich (St. Louis, MO, USA). Mueller–Hinton agar (MHA), blood agar-based (BAB) and Lowenstein–Jensen (LJ) medium were obtained from Becton Dickinson (Franklin Lakes, NJ, USA). Garlic bulbs were purchased from the local market.

### 2.1. Synthesis of Chitosan Nanoparticles

CNPs were synthesized via ionotropic gelation with TPP. Various NaCl concentrations (100–1400 mM) were used to produce nanoparticles of different sizes [29]. Stock solutions were prepared, consisting of 0.1% (*w*/*v*) low-molecular-weight chitosan (CS) in 0.01% (*v*/*v*) glacial acetic acid and 0.1% (*w*/*v*) TPP in Milli-Q water. NaCl solutions were prepared at concentrations of 100, 600, 1200, and 1400 mM. To adjust the pH, a 0.1 M NaOH solution was also prepared in Milli-Q water.

To promote nanoparticle formation, the pH of the CS solution was adjusted to 4.7–4.8 by adding 0.1 M NaOH dropwise. Subsequently, 1 mL of NaCl was added to each stock solution (CS and TPP) at the required concentration, and the mixture was agitated for one minute. Then, 3.95 mL of the TPP/NaCl mixture was added dropwise to the CS solution under constant magnetic stirring for five minutes. The samples were placed in glass vials and allowed to stand for 24 h to reach equilibrium. To remove unreacted material, the samples were purified by dialysis using membrane tubes (MWCO 12–14 kD) against distilled water for 24 h.

### 2.2. Physicochemical Properties of CNPs

The particle size distribution, polydispersity index (PDI), and Z-potential of the nanoparticles were assessed through dynamic light scattering (DLS) and electrophoretic mobility using the ZetaSizer Nano ZS (Malvern Panalytical, MA, USA). Analysis was conducted after 24 h of equilibration for all samples. Samples were appropriately diluted with deionized water. Each measurement was conducted in triplicate, and the average value of the three samples was recorded. The particle size distribution is represented by a PDI, ranging from 0 for a completely uniform dispersion to 1 for a highly heterogeneous system. Additionally, changes in nanoparticle size and surface charge were evaluated at pH levels ranging from 2 to 10.

The morphology of the CNPs was examined using Field-Emission Scanning Electron Microscopy ((FESEM) JSM-7800F Prime, JEOL, Peabody, MA, USA). To prepare the sample, 2 μL of a CNP solution with a concentration of 2 mg/mL was applied onto the surface of a 400-mesh copper–carbon grid. Subsequently, 2 μL of uranyl acetate solution (2 mg/mL) was added and left to dry. The sample was then inserted into the sample chamber and analyzed at 25 kV to capture images of the nanoparticles.

Stability studies were conducted on nanoparticle solutions stored in glass vials at 4–8 °C for 30 days. Size distributions, PDI, and pH were assessed every eight days, with samples equilibrated to room temperature before each analysis.

### 2.3. Water-Based Extraction of Garlic

To explore the antimicrobial properties of natural extracts, aqueous extractions were carried out on purple, black, and Chinese garlic bulbs (*Allium sativum*, *Allium neapolitanum*, and *Allium sphaerocephalon*, respectively). This involved macerating 5 g of each plant in 50 mL of double-distilled water. To enhance the stability of the active components within the plant, fresh garlic bulbs were first cut into 5 mm cubes and then subjected to lyophilization. Once dried, they were crushed and sieved (315 μm) to obtain a uniform powder.

### 2.4. Antimicrobial Activity Assays

The disk diffusion method was employed following the CLSI M02-A12 protocol to conduct antimicrobial tests against *Escherichia coli* (ATCC 25922), *Enterococcus faecalis* (ATCC 29212), *Staphylococcus aureus* (ATCC 25923), and *Candida albicans* (ATCC 14053) strains. Antimicrobial discs were manufactured from filter paper (Whatman No.1) cut into diameters of 6 and 8 mm and subsequently impregnated with natural extracts. Absorption proceeded until a final concentration of 300 μg per disc was attained.

For the determination of minimum inhibitory concentration (MIC) and minimum bactericidal concentration (MBC) of the CNPs, the broth microdilution method was utilized as outlined in the CLSI M07-A10 protocol.

Four nanoparticle sizes (134.6, 164.6, 216.2, and 280.0 nm) were tested against three bacteria—*S. aureus*, *E. faecalis*, *E. coli*—and one yeast, *C. albicans*. Serial microdilutions ranging from 2.0 μg/mL to 0.0625 μg/mL were performed for each nanoparticle size. Microbial cell suspensions were standardized to the 0.5 McFarland scale.

The MIC was determined as the lowest concentration of CNPs at which no visible growth was observed in the well after 24 h of incubation at 37 °C. To determine the MBC, 100 μL of the content from wells showing no visible growth were plated on MHA plates and incubated for 24 h at 37 °C. The MBC was defined as the lowest concentration that eliminated 99.9% of microbial growth. Each test was conducted in triplicate.

The agar proportion method, considered the gold standard for susceptibility testing in MTB, was employed in this assay. The protocol outlined in CLSI M24-A2 was adapted, replacing Middlebrook 7H10 agar with 5% blood agar-based (BAB). This adjustment offered a faster and more cost-effective approach while preserving the effectiveness of the conventional method and enhancing the diagnostic process.

Tests were performed using clinical strains obtained from the Department of Health in Vulnerable Populations of the Autonomous University of Baja California, identified as part of the *M. tuberculosis* complex via smear microscopy and culture in Lowenstein–Jensen (LJ) medium.

Four sizes of CNPs (116.6, 144.3, 279.1, and 364.4 nm) were tested at concentrations ranging from 100 to 400 μg per quadrant. One quadrant served as a control, containing agar medium without additives to monitor growth. A standardized cell suspension was then inoculated into each quadrant of the agar plate. All experiments were conducted in triplicate.

Growth was observed over a three-week period before colony counting [30]. Interpretation considered isolates as resistant if the colony count in the nanoparticle quadrant equaled or exceeded 1% of the count in the control quadrant.

## 3. Results

### 3.1. Synthesis and Characterization of Chitosan Nanoparticles

**Figure 1:**
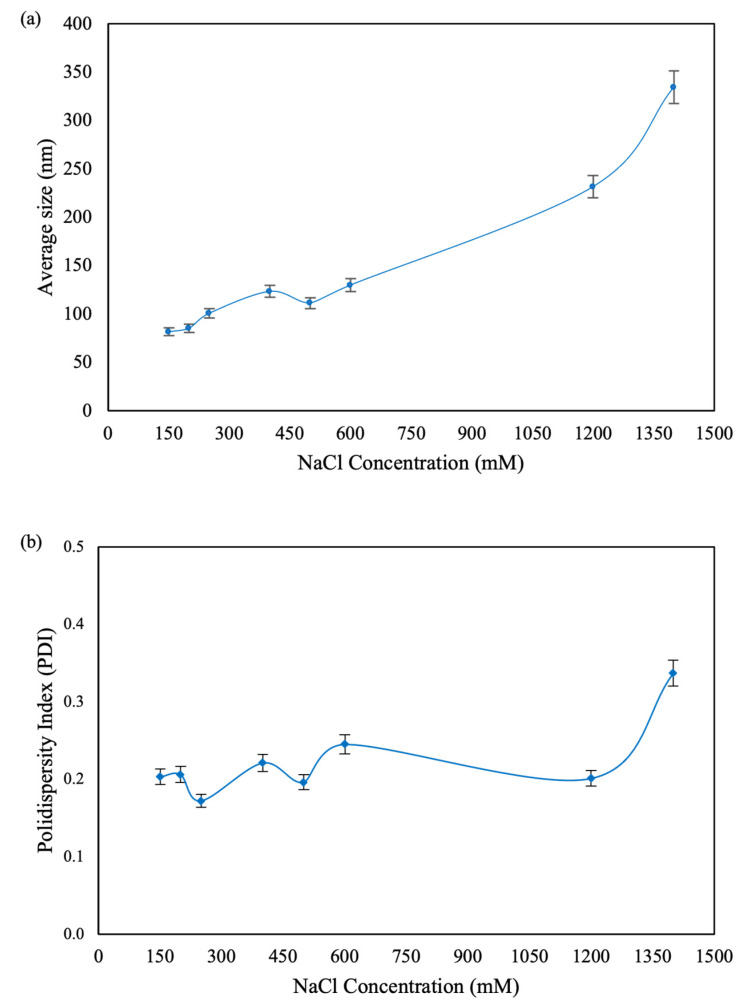
Average size and PDI values of CNPs obtained from DLS. (**a**) Effects of NaCl concentrations on the average hydrodynamic diameter. (**b**) PDI of prepared particles.

CNPs were obtained in different sizes, ranging from approximately 80 to 350 nm. The particle size variation was directly influenced by the concentration of NaCl added during the synthesis process (Figure 1), consistent with previous studies [29]. The increase in particle size with higher salt concentrations is attributed to the electrostatic repulsion caused by the added ionic strength from the monovalent salt. This results in an increased frequency of collisions in solution, which in turn leads to coagulation of sodium tripolyphosphate with the chitosan chains, forming aggregates of primary particles as the ionic strength increases [31].

#### 3.1.1. Effect of pH on Surface Charge and Size of CNPs

**Figure 2:**
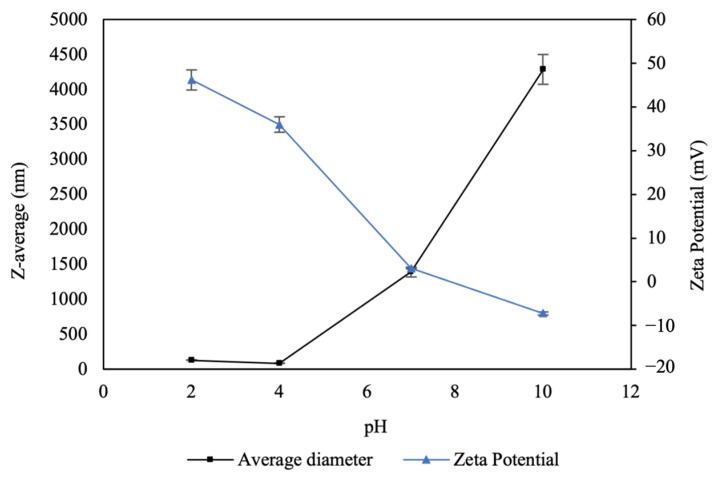
Effect of pH on particle size and surface charge of CNPs. Nanoparticles exhibit greater stability at pH levels below 7. As the pH increases the Zeta potential decreases and the size increases, indicating reduced stability.

An analysis of CNPs exposed to different pH levels was performed to examine changes in surface charge (Z-potential) and hydrodynamic diameter using DLS (Figure 2). The nanoparticle suspensions exhibited an initial pH of 4. Under these conditions, the Z-potential was positive (40 ± 3 mV), attributable to the protonation of the amino and hydroxyl groups of chitosan in an acidic medium, forming hydrogen bonds [32].

In response to increasing pH, the surface charges of the nanoparticles decreased, becoming nearly neutral at pH 7 and acquiring slightly negative charges under alkaline conditions (pH 10), with values ranging from 46.2 to −7.22 mV. Conversely, as pH increased, the sizes of the nanoparticles increased drastically with respect to the original size, reaching micrometer levels. This phenomenon could be attributed to the neutralization of the positive charges of the chitosan with the addition of NaOH, causing the formation of agglomerates.

#### 3.1.2. Morphological Analysis of CNPs

**Figure 3:**
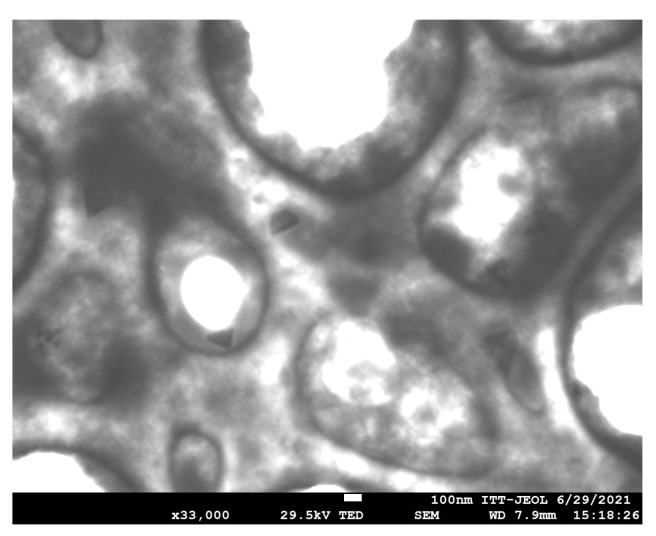
FESEM images of CNPs.

The morphology of the CNPs was examined using Field-Emission Scanning Electron Microscopy (FESEM). As shown in Figure 3, the nanoparticles exhibit low density, attributed to ionic crosslinking, which results in the formation of agglomerates.

#### 3.1.3. Stability and Size Variation of Stored Nanoparticles

Each sample was maintained at a pH of 4, which remained unchanged throughout the storage period. The smaller CNPs demonstrated good stability, retaining sizes of 87.3 ± 1.1 nm and 146.0 ± 8.0 nm. However, the larger nanoparticles exhibited greater size variation over time, reaching 219.2 ± 10.1 nm and 274.7 ± 20.7 nm after one month of storage under refrigerated conditions (4–8 °C).

### 3.2. Antimicrobial Activity

#### 3.2.1. Inhibition Tests of Natural Extracts by Disk Diffusion

The susceptibility of different *Allium* species extracts to a range of microorganisms was assessed using the disk diffusion method. Extracts from *Allium neapolitanum* (black garlic) and *Allium sativum* (purple garlic) demonstrated antimicrobial activity, producing inhibition halos against all tested microorganisms. In contrast, the extract from *Allium sphaerocephalon* (Chinese garlic) showed no inhibitory effects (Table 1).

The antimicrobial activity of *Allium sativum* has been documented by several researchers. However, it has been noted that the sulfur compounds, which are primarily responsible for this activity, exhibit limited stability, leading to a decrease in antimicrobial efficacy over time [33]. To enhance the stability of these active compounds, garlic bulbs were processed into a uniform powder as previously described at Section 2.

**Figure 4:**
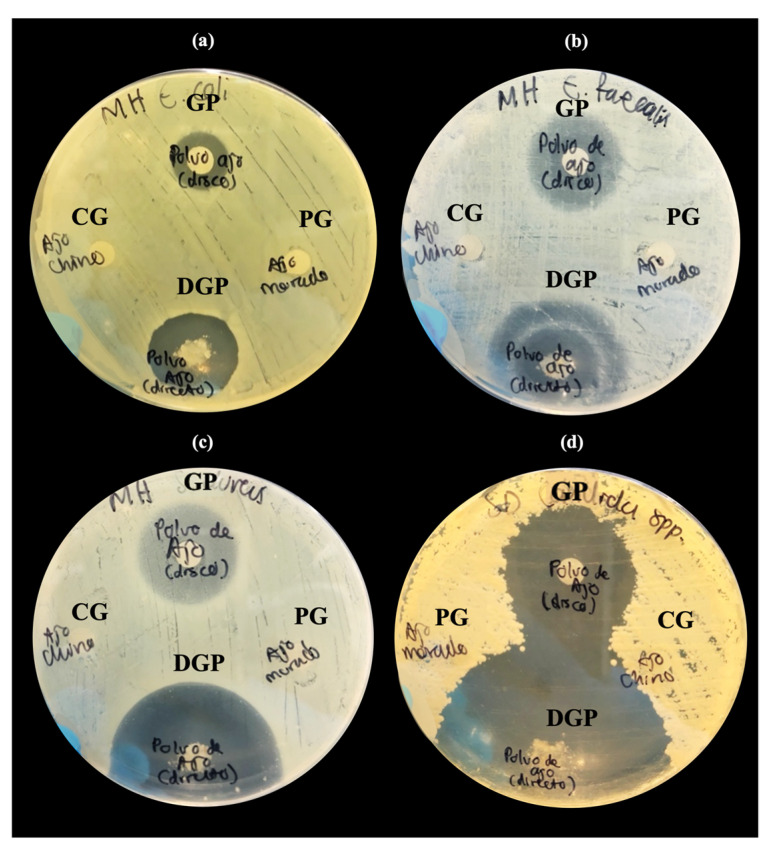
Disk diffusion susceptibility tests of *Allium sativum* powder (garlic powder, GP) and direct contact powder (DCP), as well as purple garlic (PG) and Chinese garlic (CG) aqueous extracts at 100 μg/disc on MH agar plate. Inhibition zones are shown against *E. coli* (**a**) and *E. faecalis* (**b**) demonstrating the Eagle effect, *S. aureus* (**c**), and *C. albicans* (**d**).

After obtaining the garlic powder, additional inhibition tests were conducted to verify that the antimicrobial activity remained effective following freezing and lyophilization. The inhibition zones produced were larger in diameter and achieved with a lower concentration compared to the aqueous extract (Figure 4). The most significant inhibition zone was observed against *Candida albicans*, measuring 25 mm in diameter (Table 2).

In the case of *Enterococcus faecalis*, the Eagle effect was observed (Figure 4b), which describes a paradoxical reduction in microbial death when concentrations exceed the optimal bactericidal concentration of the antibiotic. This phenomenon could present challenges in the therapeutic application of the compound [34].

#### 3.2.2. Antimicrobial Activity of CNPs Assessed by Broth Dilution Method

The antimicrobial efficacy of nanoparticles against various microorganisms was evaluated using the broth microdilution method with four nanoparticle sizes. Table 3 presents the MIC and MBC results for each microorganism.

Among the tested nanoparticles, those measuring 280 nm exhibited the highest antimicrobial activity against *E. faecalis*, with an MIC of 0.125 μg/mL and an MBC of 0.25 μg/mL. In contrast, *C. albicans* displayed the highest resistance, exhibiting growth across all dilutions tested against the 280 nm nanoparticles.

Proposed mechanisms of action for chitosan against various bacterial and fungal groups primarily focus on its polycationic nature and interactions with cell membranes. Under acidic conditions (pH < 6.5), cationic chitosan binds to phospholipids on the cell surface, disrupting essential bacterial functions [35,36,37].

#### 3.2.3. Antimycobacterial Activity of CNPs Assessed by Agar Proportion Method

Mycobacterial growth was successful in the BAB medium, allowing the counting and identification of MTB colonies. Results were classified as sensitive when no growth occurred and resistant when growth was present in the quadrant containing nanoparticles.

**Figure 5:**
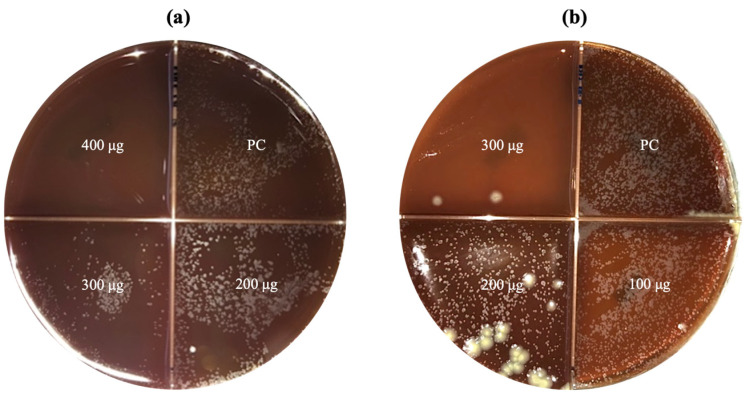
Susceptibility test using the agar proportion method in 5% blood agar against a clinical strain of *Mycobacterium tuberculosis*, where (**a**) CNP1400 (364.4 nm) completely inhibited growth at 400 μg concentration, and (**b**) CNP100 (116.6 nm) inhibited growth at 300 μg. PC (positive control) refers to the quadrant used as growth control.

MTB showed susceptibility to two nanoparticle sizes: CNP100 (116.6 nm) at 300 μg and CNP1400 (364.4 nm) at 400 μg, both of which completely inhibited growth (Figure 5). The remaining nanoparticle sizes demonstrated no inhibitory effects on mycobacteria at any concentration (Table 4).

### 3.3. Morphological Comparison of Clinical Strain Colonies of MTB

**Figure 6:**
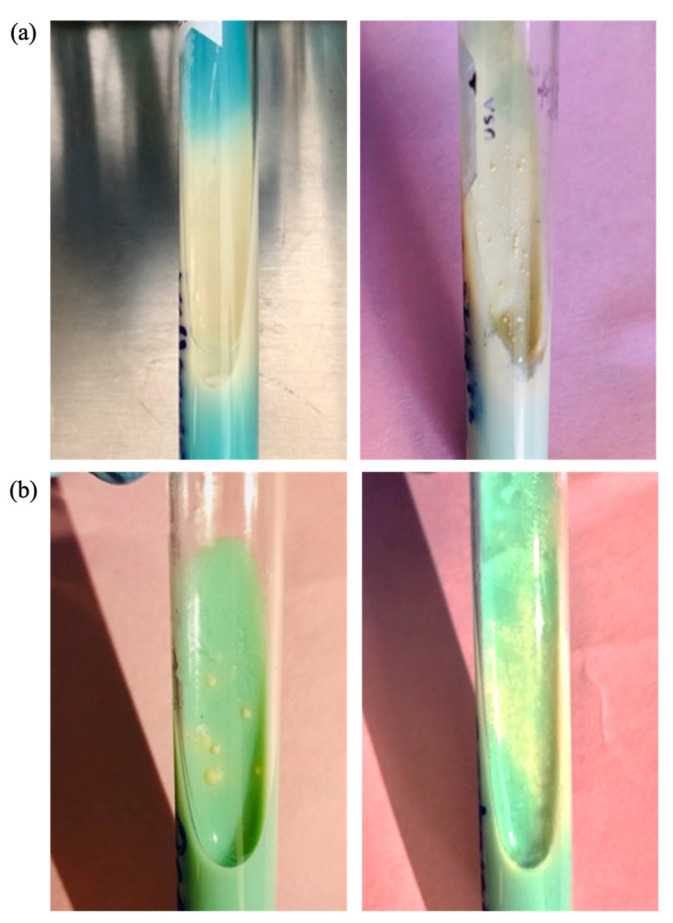
Morphological variants of *Mycobacterium tuberculosis* clinical isolates. Monolayer growth (**a**) and typical growth (**b**).

When cultured on LJ medium, clinical isolates of MTB displayed distinct morphological variations. Some colonies exhibited typical growth characteristics with rough, yellowish-white appearance, while others displayed smooth, monolayer growth (Figure 6).

This occurrence has been documented previously and attributed to the absence of glycolipid trehalose 6,6-dimycolate (TDM) in the bacillus cell wall. TDM typically imparts a rough texture to colonies and its absence is linked to reduced virulence of the mycobacterium. The potentially heterogeneous population of intracellular bacilli exploits this lower virulence state to sustain long-term coexistence within the human host [38,39].

## 4. Discussion

The antimicrobial activity of CNPs against various microorganisms, as demonstrated by their MIC and MBC values, aligns with previous studies highlighting their efficacy due to their polycationic nature. This property allows CNPs to interact with microbial cell membranes, altering their integrity and leading to inhibition or eradication of the pathogens evaluated [35,36,37].

The observed variation in antimicrobial efficacy among different CNP sizes underscores the importance of nanoparticle size in determining their effectiveness. Smaller nanoparticles, such as CNP100, exhibited greater potency against certain bacteria and fungi compared to larger sizes. This could be attributed to better penetration and interaction at the cellular level, enhancing their bactericidal or fungicidal effects [40]. However, larger nanoparticles, such as CNP1400, were able to inhibit MTB growth. This may be attributed to a larger surface area and charge density for interacting with microbial membranes [41]. This size-dependent effectiveness suggests that nanoparticle optimization could play a crucial role in maximizing antimicrobial efficacy.

Moreover, exploration of the antimicrobial properties of *Allium sativum* revealed significant potential, mainly against the yeast *C. albicans*. Its antimicrobial properties have been widely documented, and its efficacy is attributed to the presence of sulfur compounds, mainly allicin [22]. Our findings corroborate these reports, demonstrating that both aqueous extracts and garlic powder exhibit substantial antimicrobial activity against several microorganisms.

The improved stability of the active compounds and the antimicrobial efficacy of garlic, achieved by freeze-drying, ensures the preservation of the natural product, offering a viable alternative to conventional antimicrobial agents.

The combination of *Allium sativum* extracts and CNPs presents a promising avenue for the development of novel antimicrobial agents, thus addressing the pressing problem of antimicrobial resistance. Furthermore, the natural origin of these compounds aligns with the growing demand for sustainable and environmentally friendly antimicrobial solutions.

However, the use of materials for biomedical applications raises biocompatibility concerns [42]. CNPs have been widely studied for their use in drug delivery systems due to their biocompatibility, biodegradability, and non-toxicity. Studies have shown that CNPs can be safely used to deliver a variety of drugs, including anticancer agents, antibiotics, and vaccines. They are well-tolerated by human cells and can enhance the therapeutic efficacy of encapsulated drugs by improving their stability and release profiles [35]. Additionally, CNPs promote wound healing through their hemostatic properties and ability to enhance cellular responses, improving tissue repair by promoting the proliferation and migration of fibroblasts and keratinocytes, essential for tissue repair [43].

In tissue engineering, CNPs have been used to create biocompatible scaffolds that support the growth and differentiation of cells and naturally degrade in the body, making them ideal for regenerative medicine applications [44]. Generally, CNPs exhibit low cytotoxicity and are safe for biomedical use. In vitro and in vivo studies indicate that these nanoparticles do not cause significant adverse effects on cell viability, proliferation, or differentiation, although toxicity can vary depending on the size and concentration [45,46].

On the other hand, garlic extracts and derivatives have beneficial properties and are generally safe; however, at higher concentrations, they may cause significant adverse effects on cellular responses [47,48,49].

In the current study, we proved the antimicrobial activity of chitosan nanoparticles with size variations, as well as that of garlic extract and powder. To fully apply these materials as antimicrobials, they must be formulated to target infected cells (e.g., inhalation powder). To take full advantage of the therapeutic potential of these natural products, comprehensive cytotoxicity studies and in vivo evaluations of the final formulations are essential to ensure the safety and efficacy of these compounds in clinical settings.

Regarding the morphological variations observed among clinical isolates of MTB, they are consistent with previous reports associating morphology with strain virulence [38]. Rough colonies, related to the presence of the glycolipid trehalose 6,6-dimicolate (TDM) in the cell wall, indicate greater virulence and robust survival of the pathogen. In contrast, smooth colonies without TDM suggest a less virulent state linked to lower pathogenicity and persistence in the host.

Understanding these morphological variations is crucial to unveiling MTB adaptive strategies within the host environment. The heterogeneous MTB population with different virulence states could take advantage of the less virulent phenotype to evade immune detection and establish prolonged infections in human hosts. This adaptive strategy brings out the complex interplay between MTB and the host immune response, which influences disease progression and treatment outcomes [50].

Future research could explore the genetic and molecular mechanisms behind morphological variations in MTB colonies. Examining the pathways that regulate TDM biosynthesis and its impact on virulence could reveal new therapeutic targets for tuberculosis. In addition, analyzing the relationship between colony morphology on disease severity and response to treatment could provide valuable information for personalized treatment strategies.

## 5. Conclusions

CNPs and *Allium sativum* demonstrated significant efficacy against a spectrum of microorganisms, encompassing Gram-positive and Gram-negative bacteria, fungi, and even the challenging pathogen *Mycobacterium tuberculosis*. Particularly noteworthy was the extract of *Allium sativum,* which showed remarkable efficacy against *Candida albicans*. These findings pave the way for future research aimed at optimizing natural antimicrobial agents, ultimately contributing to the development of safe, effective, and sustainable treatments for infectious diseases.

Further research into optimizing formulations could lead to the development of valuable alternatives or supplements to conventional antimicrobial therapies. However, it is important to emphasize the need for cytotoxicity studies and in vivo evaluations to assess the safety of these compounds in clinical applications.

## Figures and Tables

**Table 1:** Inhibition zones of *Allium* species extracts against various microorganisms.

Organism	Diameter of Zone of Inhibition (mm)		
	*Allium sativum* (300 μg)	*Allium neapolitanum* (300 μg)	*Allium sphaerocephalon* (300 μg)
*E. coli* ATCC 25922	8	5	0
*E. faecalis* ATCC 29212	8	5	0
*S. aureus* ATCC 25923	12	9	0
*C. albicans* ATCC 14053	10	7	0

**Table 2:** Inhibition zone diameters of *Allium sativum* lyophilized powder by disk diffusion and direct contact with agar.

Organism	Diameter of Zone of Inhibition (mm)	
	*Allium sativum* (100 μg)	*Allium sativum* (Direct Contact Testing of Powder)
*E. coli* ATCC 25922	9	13
*E. faecalis* ATCC 29212	12	14
*S. aureus* ATCC 25923	12	21
*C. albicans* ATCC 14053	15	25

**Table 3:** MIC and MBC (μg/mL) of CNPs against various microorganisms.

CNPs	Size (nm)	MIC and MBC (μg/mL) of Nanoparticles against Organisms							
		*E. coli*		*E. faecalis*		*S. aureus*	*C. albicans*		
		MIC	MBC	MIC	MBC	MIC	MBC	MIC	MBC
CNP100	134.6	0.25	0.5	0.5	1	0.25	0.5	2	-
CNP600	164.6	2	2	2	2	2	2	2	-
CNP1200	216.2	1	1	0.125	0.25	2	2	0.25	0.5
CNP1400	280.0	0.125	0.25	0.125	0.5	2	2	-	-

**Table 4:** Critical concentrations of CNPs against *M. tuberculosis* clinical strain.

CNPs	Size (nm)	Nanoparticle Concentration (μg)			
		100	200	300	400
CNP100	116.6	R	R	S	NT
CNP600	144.3	NT	R	R	R
CNP1200	279.1	R	R	R	NT
CNP1400	364.4	NT	R	R	S

NT: non-tested, R: resistant, S: susceptible.

## Data Availability

Data are contained within the article.

## Abbreviations

AMR	Antimicrobial resistance
BAB	Blood agar- based
CNPs	Chitosan nanoparticles
CS	Chitosan
DLS	Dynamic light scattering
LJ	Lowenstein–Jensen
MBC	Minimum bactericidal concentration
MDR-TB	Multidrug-resistant tuberculosis
MHA	Mueller–Hinton agar
MIC	Minimum inhibitory concentration
MTB	Mycobacterium tuberculosis
MWCO	Molecular weight cut-off
NaCl	Sodium chloride
NaOH	Sodium hydroxide
PDI	Polydispersity index
SEM	Scanning electron microscopy
TB	Tuberculosis
TMD	Glycolipid trehalose 6,6-dimycolate
TPP	Sodium pentabasic tripolyphosphate
XDR-TB	Extremely resistant tuberculosis
